# Obstacles and facilitators of cancer-related dyadic efficacy experienced by couples coping with non-metastatic cancers

**DOI:** 10.3389/fpsyg.2023.949443

**Published:** 2023-06-08

**Authors:** Danielle C. Brosseau, Sandra Peláez, Bethsheba Ananng, Annett Körner

**Affiliations:** ^1^Department of Psychology, The King’s University, Edmonton, AB, Canada; ^2^Department of Educational and Counselling Psychology, McGill University, Montreal, QC, Canada; ^3^École de kinésiologie et des sciences de l'activité physique (EKSAP), Faculté de Médecine, Université de Montréal, Montreal, QC, Canada; ^4^Lady Davis Institute, Jewish General Hospital, Montreal, QC, Canada; ^5^Department of Oncology, McGill University, Montreal, QC, Canada; ^6^Louise Granofsky Psychosocial Oncology Program, Segal Cancer Center, Montreal, QC, Canada; ^7^Psychosocial Oncology Program, McGill University Health Centre, Montreal, QC, Canada

**Keywords:** cancer, couples, dyadic efficacy, reflexive thematic analysis, focus groups, qualitative research methods, psychosocial

## Abstract

**Introduction:**

Cancer-related dyadic efficacy is an individual’s confidence to work together with a partner to conjointly manage the effects of cancer and its treatment. In other health contexts, higher levels of dyadic efficacy have been associated with fewer symptoms of psychological distress and higher ratings of relationship satisfaction. The aim of the current study was to explore patient and partner perspectives on what obstructs and facilitates cancer-related dyadic efficacy.

**Methods:**

These aims were accomplished through a secondary analysis of data collected as a part of a collective qualitative case study. Participants (*N* = 17 participants) were patients undergoing treatment or recently completed treatment (within 6 months) for a non-metastatic cancer and their partners. To enable in-depth discussions among participants, data was collected through five focus groups. Participants described obstacles and facilitators of dyadic efficacy as dimensions of a common influence. Consistent with these descriptions, reflexive thematic analysis was used to identify influences on cancer-related dyadic efficacy and their subsequent obstructive and facilitative dimensions.

**Results:**

Four main categories of influence with the potential to obstruct or facilitate cancer-related dyadic efficacy were identified along with their subthemes: appraisals of the couple relationship (quality and togetherness), communication (pattern and interest in information), coping (strategy and evaluation), and responses to change (in tasks and roles and sex life). Eight obstructive and seven facilitative dimensions of these subthemes were described.Discussion: This first analysis of obstacles and facilitators of couples’ cancer-related dyadic efficacy capitalized on the experiential expertise of individuals with cancer and their partners. These thematic results are instructive for the design of dyadic efficacy-enhancing interventions for couples coping with cancer.

## Introduction

In addition to the obvious physical effects, cancer and its subsequent treatment commonly affect the psychological, social, and spiritual well-being of the individual diagnosed and their family members (Stenberg et al., 2010; Carlson et al., 2011; Caruso et al., 2017; Bubis et al., 2018). Approximately 33 % of those diagnosed with cancer will also require support for a co-occurring mental health concern (Singer, 2018). The multiple effects of cancer on both the patient and their partner have led researchers to conceptualize cancer as a dyadic stressor (Bodenmann, 2005). An understanding of cancer as a dyadic stressor is also an important acknowledgement of the elevated levels of psychological distress that can be experienced by the individual diagnosed with cancer and his or her partner (Hagedoorn et al., 2008; Kuenzler et al., 2011; Moser et al., 2013).

Viewing the couple as the unit of analysis and accounting for the interactions that occur within the interdependent system of the couple provides advanced insight into couples’ psychological distress and coping following a cancer diagnosis (Kayser et al., 2007; Badr et al., 2010; Traa et al., 2014; Regan et al., 2015; Jacobs et al., 2017). Looking outside of oncology, health researchers have applied a systemic approach to the study of efficacy expectations, examining what they termed, dyadic efficacy (Sterba et al., 2007, 2011). Dyadic efficacy extends from Albert Bandura’s social cognitive theory, offering a dyadic counterpart to the individually-focused construct of self-efficacy (Bandura, 1977). Cancer-related dyadic efficacy is an individual’s judgment of his or her confidence to conjointly manage the effects of cancer and its treatment together with a partner.

The interactions of patients’ and partners’ ways of coping with cancer influence each individual’s psychological health and their relationship satisfaction (Berg and Upchurch, 2007; Berg et al., 2008; Badr et al., 2010; Rottmann et al., 2015). Dyadic efficacy represents a couples’ appraisal of their joint coping capability and has the potential to be an important personal resource to identify and enhance among patients with cancer and their partners. In his early writings on self-efficacy, Bandura asserted that an individual’s belief that he or she could complete a behavior (efficacy expectation) greatly influenced the probability that the individual would enact the behavior and would sustain the behavior in the face of obstacles (Bandura, 1977). Provided Bandura’s assertions regarding self-efficacy extend to dyadic efficacy, patients’ and partners’ dyadic efficacy expectations may be a powerful tool for the propulsion and perseverance of beneficial joint coping actions. With these expectations in mind, it becomes essential to better understand what might impede or enhance cancer-related dyadic efficacy.

In the initial research on dyadic efficacy among couples managing one partner’s rheumatoid arthritis, higher dyadic efficacy was associated with fewer depressive symptoms and higher ratings of relationship satisfaction and quality for both women with rheumatoid arthritis and their husbands (Sterba et al., 2007). Similarly, dyadic efficacy for smoking cessation was positively associated with relationship satisfaction and also predictive of support behaviors and dyadic coping (Sterba et al., 2011). Although no identification of facilitators or obstacles to dyadic efficacy were found, these associations between dyadic efficacy, psychological distress and relational factors may foreshadow the content of facilitative and obstructive influences on dyadic efficacy.

## The present study

This study is part of a larger mixed-methods endeavor that, to our knowledge, was the first to examine dyadic efficacy in the cancer context (Brosseau et al., 2021, 2023). The primary data set used here was first collected to facilitate consultation with lay experts (individuals with cancer and their partners) regarding the conceptualization of cancer-related dyadic efficacy and the identification of content domains for assessment. In this foundational research, thematic analysis was used to describe three main qualities of cancer-related dyadic efficacy (it is multidimensional, consistent with established relational functioning and distinct from self-efficacy) and three main themes encompassing eight content domains that participants described to be essential for the assessment of cancer-related dyadic efficacy. These themes and domains reflected dyadic efficacy for managing: (a) illness intrusions related to the patients’ physical experience, social life, couple life, the medical system, and ongoing responsibilities, (b) emotional responses of the patient and the partner, and (c) communication and care for children (Brosseau et al., 2023). Expanding on this initial work, the objective of the present study was to construct themes that reflected what facilitated or obstructed patients’ and partners’ cancer-related dyadic efficacy. The research question guiding the inquiry was: what helps or hinders couples’ confidence to cope with cancer-related challenges together as a unit?

## Materials and methods

### Study design

This study presents the results of a secondary analysis of focus group data collected within the exploratory phase of the aforementioned scale development study (Brosseau et al., 2021). Long-Sutehall et al. (2010) recommend that researchers consider the appropriateness of secondary qualitative data analysis, including a consideration that the research questions are appropriate to the primary data. The impetus for this secondary data analysis emerged during thematic analyses of the primary data set. Participants’ discussions of cancer-related dyadic efficacy and its measurement naturally extended into descriptions of what enhanced or hindered their confidence to manage cancer-related challenges together as a unit.

The process of eliciting participants’ insights on cancer-related dyadic efficacy was guided by a collective qualitative case study design, which involved an in-depth analysis of multiple bounded systems (Stake, 2005). The boundaries set around the selection of cases are further detailed in the participants section below. Rooted in the social constructivist paradigm, knowledge was understood to be co–constructed through the dynamic interactions that occurred among the participants and the researchers (Stake, 1995; Gergen, 2009). With collective academic and clinical expertise in psychosocial oncology, psychology and qualitative research methods, the researchers were outsiders in relation to the participants in this study. As outsiders, the research team was cognizant of the need to continuously reflect on our own assumptions, carefully considering interpretations of the data that prioritized participants’ voices.

### Participants

Patients were eligible if they were currently receiving treatment or had recently completed treatment (within 6 months) for a non-metastatic cancer and were involved in a committed intimate relationship of at least 1 year (e.g., dating, common law, married). Partners of patients meeting these medical criteria were also invited to participate. All participants were (a) able to read and comprehend English, (b) 18 years of age or older, and (c) able to provide informed consent. The participation of complete dyads was sought but, to reduce barriers to participation, a patient or a partner was eligible to participate without the other member of the dyad. Patients diagnosed with metastatic disease were excluded in an effort to focus the discussion on confidence for managing the challenges of diagnosis and active treatment and to limit the heterogeneity of the sample.

### Procedures

This study was approved by the research ethics committee of the Jewish General Hospital, Montréal, Canada (protocol #14–078). Convenience sampling was used to recruit eligible patients and partners. Recruitment was conducted through advertisement (paper and online) of the study at a large urban cancer centre and para-support programs in Montréal, Canada. In an effort to increase the diversity of the sample, referring health care providers (e.g., nurses, support staff) were encouraged to refer couples who reported ease working together to cope with cancer as well as those that reported great difficulty facing cancer-related challenges together as a unit.

Focus groups were chosen because this method facilitates the generation and refinement of ideas amongst participants and between participants and the researchers (Morgan, 1996). Based on recommendations in the literature, it was anticipated that conducting three to five focus groups would enable response consistency given the confined focus on cancer-related dyadic efficacy (Burrows and Kendall, 1997; Krueger and Casey, 2009). Small focus groups (*n* = 5–7 participants) were planned to aid the researchers’ desire to elicit in-depth discussions among the participants at the couple and group level (Liamputtong, 2011). Beyond the bounds of each case, focus group composition was heterogeneous. The semi-structured focus groups ranged from 45–97 min ( $\bar{X}$ = 75 min). Focus groups began with an introduction and welcome from the researchers, followed by the completion of informed consent. Participants were then invited to introduce themselves and proceeded to complete and evaluate newly generated questionnaire items querying dyadic efficacy. This initial procedure was used for the broader scale development study. The researchers then facilitated a discussion of dyadic efficacy, informed by a topic guide that centered the discussion on patients’ and partners’ (a) descriptions of cancer-related dyadic efficacy, (b) perspectives on the types of challenges encountered, and (c) experiences coping with these cancer-related challenges together. For example, participants were asked about their conjoint coping efforts generally (In what ways do you and your partner work together to cope with cancer?) and more behaviorally (What tasks do you and your partner manage together as a part of coping with cancer?). Participants received a 20-dollar reimbursement for transportation or parking costs. Author DB led each group with the assistance of a co-moderator and a participant observer. Data collection using focus groups has been referred to as a process of listening in or eavesdropping (Barbour, 2007). DB adopted this stance to encourage participants’ engagement with each other as they unraveled their own and each other’s perspectives on dyadic efficacy. The co-moderator was minimally involved in querying participants’ responses and primarily responsible for the organization of the group including recordings, timing, and note–taking (Krueger and Casey, 2009). Focus groups have been criticized for masking the effects of agreement or disagreement among group members in favour of the most dominant voices. The small size of the focus groups limited the potential for differing opinions to be masked because the facilitator was reasonably able to follow-up and inquire about the extent to which an idea resonated with the group more broadly.

### Data analysis

All focus groups were audio-recorded and transcribed verbatim. Each transcript was examined by an independent second reviewer (a volunteer research assistant) and DB for consistency with the recording. With the exception of grammatical adjustments, the participant quotes presented in this report are verbatim (Poland, 2001). Data available for analysis included the data derived from the focus group transcripts, moderator and co-moderator notes and impressions. A reflexive thematic data analysis was conducted using Braun and Clarke’s (2006, 2019) guidelines and included immersion in the data, generating initial codes, searching, reviewing and developing themes. An inductive approach to theme construction was used whereby the researchers sought to generate key themes that captured the diversity of what influenced participants’ confidence to cope with cancer conjointly. MAXQDA (VERBI GmbH, Germany, version 11) was used to aid the analysis. DB familiarized herself with the data to the point of immersion through multiple readings of the transcripts and supplemental data with a focus on identifying what influenced participants’ cancer-related dyadic efficacy. It became evident early in the immersion process that a given influence on dyadic efficacy did not act as a facilitator or an obstacle but that many influences on couples’ confidence had facilitative or obstructive dimensions. In keeping with our data-driven approach, the focus of the coding process shifted to capture participants’ descriptions of facilitative and obstructive dimensions of the same influence on dyadic efficacy. Journaling was used to denote potentially important codes, emerging theme ideas, and insights related to the similarities across influences. An initial list of what were later-termed subthemes was developed, discussed, and reviewed conjointly with SP. Using these identified subthemes, DB and SA independently coded each transcript, engaged in reflexive discussions regarding the meaning ascribed to a given coded segment, returned to the data, and redefined or expanded the subthemes to ensure they captured the essential aspects of the participants’ descriptions. During theme construction, the researchers also reviewed and discussed similarities across themes which led to the construction of theme categories that functioned to group similar influences on dyadic efficacy together in meaningful clusters. Nowell et al. (2017) outline several strategies for enhancing the credibility of thematic analysis including the use of researcher triangulation. Researcher triangulation was used in this study during the development of the initial codes (DB and SP), throughout code development (DB and SA) and when reviewing the theme categories and their subthemes (DB, SA and SP).

## Results

### Participant characteristics

Participants (*N* = 17) included six patient–partner dyads, four patients and one partner who participated alone (see Table 1 for participant characteristics). Five focus groups were conducted with fewer participants then expected (*n* = 2–5 participants per group). Issues of coordinating participants’ schedules, illness demands, and inclement weather limited the size of the focus groups and led to what Krueger and Casey (2009) termed, mini-focus groups. Reasons for participating alone included (a) their own or their partner’s preference, (b) limited language abilities in English, or (c) work or childcare responsibilities. Both female (*n* = 6) and male (*n* = 4) individuals with cancer participated. Patients were on average 60 years old (range = 44–72 years), in relationships ranging from three to 48 years in duration ( $\bar{X\;}$ = 22 years), and heterogeneous with respect to the type of cancer diagnosed. Partners were female (*n* = 4), male (*n* = 2) or non-binary (*n* = 1), an average of 51 years of age (range 25–72 years) and in relationships ranging from five to 48 years in duration ( $\bar{X}=$ 26 years). Ten of the 11 distinct dyads represented heterosexual partnerships (one dyad included a female and a non-binary individual).

**Table 1:** Patient and partner characteristics (*N* = 17).

Dyad represented	Pseudonym	Role	Sex	Age range (years)	Relationship length (years)	Tumour site^a^	Stage of cancer^a^	Time since diagnosis^a^
1	Kelly	Patient	Female	40–49	8	Breast	1	6 months – 1 year
	Felix	Partner	Male	50–59	8			
2	Joanne	Patient	Female	50–59	11	Thyroid	2	Over 1 year
	Scott	Partner	Non-binary	30–39	11			
3	Luc	Patient	Male	60–69	38	Myeloma	1	Over 1 year
	Alice	Partner	Female	60–69	38			
4	Francis	Patient	Male	60–69	32	Palate	1	6 months – 1 year
	Ines	Partner	Female	50–59	32			
5	Gina	Patient	Female	70–79	48	Breast	1	Over 1 year
	Barry	Partner	Male	70–79	48			
6	Roy	Patient	Male	60–69	40	Multiple Myeloma	3	Over 1 year
	Fiona	Partner	Female	50–59	40			
7	Stephanie	Partner	Female	20–29	5	Lymphoma	Unknown	3–6 months
8	Louise	Patient	Female	40–49	12	Breast	3	Over 1 year
9	Tina	Patient	Female	50–59	12	Breast	1	6 months – 1 year
10	John	Patient	Male	70–79	3	Prostate	Unknown	Over 1 year
11	Sharon	Patient	Female	70–79	12	Multiple myeloma	Unknown	Over 1 year

a All medical data was self-reported by the study participant.

Medical data were not listed for partner participants with the following exception. Medical information corresponding to the patient was provided for the partner who participated alone.

### Obstacles and facilitators of cancer-related dyadic efficacy

Four main themes of influence and eight subthemes with the potential to facilitate or obstruct cancer-related dyadic efficacy were identified, namely cancer-related dyadic efficacy was influenced by: (a) appraisals couples made about the quality of their relationship and the meanings attributed to togetherness, (b) individual and dyadic communication patterns and preferences for cancer-related information, (c) coping strategies used and evaluations made about the chosen strategy, and (d) how changes to the division of everyday tasks and the couples’ sex life were managed. The facilitative and obstructive dimensions of these factors are presented in Table 2 and described in the following paragraphs. To protect the anonymity of participants, all names used in this paper are pseudonyms.

**Table 2:** Influences that facilitate and obstruct patients’ and partners’ cancer-related dyadic efficacy.

Category	Subtheme	Facilitator	Obstacle
A. Appraisals couples made about the quality of their relationship and the meanings attributed to togetherness			
A1	Quality of the couple relationship	Stable couple relationship	Weak couple relationship
A2	Togetherness	Valued togetherness	Devalued togetherness
B. Individual and dyadic communication patterns and preferences for cancer-related information			
B1	Communication pattern	Congruent pattern	Incongruent pattern Lack of communication
B2	Interest in cancer-related information	Shared interest	Disinterest
C. Coping strategies used and evaluations made about the chosen strategy			
C1	Dominant coping strategy	–	Avoidant coping
C2	Evaluation of partner’s coping strategies	Acceptance of partner’s coping	Disapproval of partner’s coping
D. How changes to the division of everyday tasks and the couples’ sex life were managed			
D1	Tasks and roles	Flexibility	Lack of awareness
D2	Sex life	Patience	–

### A: Appraisals couples made about the quality of their relationship and the meanings attributed to togetherness

The confidence patients and partners had in their joint ability to manage adverse experiences associated with cancer and its treatment was influenced by appraisals of (a) the quality of the couple relationship and (b) togetherness.

#### A1: Stable versus weak couple relationship

The functioning of the couple relationship prior to the cancer diagnosis was viewed as a “baseline” (Louise, woman with breast cancer) or “foundation” (Kelly, woman with breast cancer) for couples’ confidence to respond to cancer-related challenges together. Appraisal of a stable or weak pre-existing relationship was presented as a facilitator or an obstacle to dyadic efficacy. While these participants did not overtly label their own relationship to be weak, they instead expressed doubts about whether those with a weak pre-existing relationship could withstand the stress a cancer diagnosis imposes on the couple relationship. This exemplary quote illustrates this idea:

I find that if the relationship was weak, we wouldn’t have made it. I mean the fact that we already had a stable foundation as a couple—we were respectful towards each other and we love each other unconditionally. When this [cancer] happened, there was no question just to love each other and to go through it together (Kelly, woman with breast cancer).

#### A2: Valued versus devalued togetherness

Dyadic efficacy was described as being enhanced or hindered by the appraisals patients and partners made which valued or devalued the experience of being together. Participants described togetherness with varying emphases on both symbolic and actual accounts of togetherness. Those with a more practical focus, gained confidence from facing challenges and approaching tasks together with their partner (e.g., doctor’s appointments, treatments, leisure time). Roy and Fiona stated: “We always did everything together” (Roy, man with multiple myeloma); “Even when we are tired and we do not feel like talking—just sitting together on the couch and holding his hand was soothing for us” (Fiona, partner). The act of being present or simply being partnered was referred to as being beneficial, regardless of the specific behaviors or actions of the other person. As John (man with prostate cancer) described: “being aware of [my] partner’s love” was itself sustaining. For some, like John, the meaning drawn from being together provided the very reason for enduring anticipated difficulties of cancer treatment and was very focused on a symbolic feeling of togetherness. For example:

She helps me a lot, not because she did something. She helps me because I love her and the happiness I have because I love her. This helps me a lot during my treatments and still today (John, man with prostate cancer).

In contrast, dyadic efficacy was hindered when either a patient or a partner appraised togetherness as having little or no additional value for managing cancer-related challenges. For these participants, togetherness was described as unneeded, ineffective, or even a waste of time. This obstacle was well-captured in the following participant statement:

He took me to my appointment, but I told him ‘You know what, don’t come with me. I’d rather you just go get a coffee’. I had to wrap my head around it first because if I don’t do that, I’m not going to be able to cope. I knew it was going to be bad news. And I just said that if I had to worry about his emotions, then I’m not going to be able to deal with it. You know, if you cry, you cry for 5 minutes by yourself, but if you cry and you see someone else cry, it’s just back and forth and it’s never going to stop. So that’s why to me, I kind of had to… let me get through my thing, take the shot. But if we had done it together, I think it would’ve been too hard for me (Sharon, woman with multiple myeloma).

Dyadic efficacy was also obstructed when participants believed that coping together would make their coping more difficult or add additional issues to be attended. Tina (woman with breast cancer) noted: “In treatment, I definitely did not want him there, because all he would do is run around, you know, being… feeling impotent and I’d have to take care of him.” Likewise, when a participant felt that his or her experience could not be understood by his or her partner, togetherness was devalued. As Luc (man with myeloma) mentioned: “I would not put something on her that I knew she could not relate to.”

For some couples, descriptions that devalued togetherness were expressed alongside events appraised to be manageable and only minimally threatening. In contrast, those that valued togetherness, drew strength from being together regardless of the perceived difficulty of the situation. Notably, those who devalued togetherness focused on very practical aspects of being together (i.e., attending appointments, leisure time) without addressing the more symbolic perspective.

### B: Individual and dyadic communication patterns and preferences for cancer-related information

Cancer-related dyadic efficacy was influenced by patients’ and partners’ (a) communication patterns and (b) interest in cancer-related information.

#### B1: Congruent versus incongruent communication patterns and lack of communication

The extent to which communication preferences were evaluated by patients and partners to be congruent influenced confidence to jointly manage challenges. Acceptance of the communication pattern was more important than the extent to which a patient’s or a partner’s communication pattern was more open or restricted. For the purposes of this depiction, communication that was described as candid and vulnerable was labeled open, whereas communication that was described to have limits with respect to topics or depth was labeled restricted. Open communication facilitated dyadic efficacy when preferred by both members of the dyad. At times, open communication also included processing and discussing experiences, seeking clarity about their own experiences, requesting advice or seeking an alternative perspective. Kelly (woman with breast cancer), said this:

We have this rule that we have to respect: we work together, if I’m not nice or something, he has to let me know. If I don’t do well, he’ll tell me, ‘You know Kelly you’re not doing too well today. You wouldn’t do that on a normal day’.

The congruence of communication preferences between partners facilitated dyadic efficacy even when there was a dyadic preference for more restricted communication. Luc and Alice both felt satisfied with their restricted communication pattern that was largely limited to the discussion of practical issues: “I come home at night and it’s not something that we talk about. We only talk about when we see the doctor and what we are going to do and what we should do” (Luc, man with myeloma). Dyadic efficacy was obstructed when the couple differed in their preferred communication pattern. Open communication was experienced as overwhelming and an additional challenge to contend with for individuals that preferred more restricted communication.

A lack of communication reflected the pattern of those who reported that cancer was “not something [they would] talk about” (Roy, man with multiple myeloma). Naturally, when one member of the dyad adopted a preference for this communication pattern, the other member reported feeling blocked in their ability to work together. Put simply: “you cannot manage symptoms together if he does not tell you that he’s in pain” (Fiona, partner). Two main explanations were given when an individual expressed a lack of desire to talk about cancer: (a) to protect one’s partner and/or (b) a history of keeping personal experiences to oneself. Participants who restricted their cancer-related communication in an effort to protect their partner, reported a belief that increased communication would burden their partner or increase their partner’s fear or worry. Those who preferred or experienced a partner’s lack of communication described the restriction as an obstacle to their ability to cope together with their partner due, in part, to increased isolation:

I didn’t want to talk to him about it because he’s not interested and I don’t want to scare him. There are things that I know that I would want to tell him but I can’t so I sort of lock myself in the garage and I would cry by myself (Stephanie, partner).

Others shared that their tendency to keep thoughts, emotions and experiences to themselves was a long-time preference and cited little benefits to making their internal world known. Descriptions included a pattern of “keeping things to [myself]” (Louise, woman with multiple myeloma), “keeping things inside” (Roy, man with multiple myeloma), or simply “not sharing” (Fiona, partner). One couple described their experience of communication as follows: “When I ask him how he’s feelings, he will not tell me. [He’ll say,] ‘I’m okay, I’m okay’” (Fiona, partner). Roy replied, “I’ve always been like that. I do not complain. If I get a pain, I do not complain. I never complain. Why complain? Your pain is alive anyways whether you complain or not” (Roy, man with multiple myeloma). Roy and Fiona’s exchange is representative of the perspective of others who also saw little benefit to informing their partner about their symptoms or emotions. Fiona’s exchange was littered with frustration which was a typical experience for those whose partner preferred not to talk about cancer.

#### B2: Shared interest versus disinterest in cancer-related information

Regardless of personal information-seeking styles, patients and partners who expressed a shared interest in cancer-related information increased confidence to process informational challenges together. For example, one couple described their approach to medical information as follows:

She had been doing all the homework, she had been reading online (Barry, partner).

[I went] from not knowing what a mastectomy is [partner laughs] to finding out the type of cancer and really researching it… I’ve never had any interest in that kind of thing and all of a sudden, I was very knowledgeable. I wanted to know what was going on. So, it’s been good for me (Gina, woman with breast cancer).

Yeah, it’s been good for the both of us because we’ve been able to talk about it (Barry, partner).

Patients and partners reported that information about cancer became less intimidating and overwhelming when they were able to share it with their partner.

He would not be the type to be able to go on the internet to get information. For him it was always about information overload so I fed him the information. So, in that respect it was good because then I kind of controlled my own treatments but we did it as a team in a sense that I always told him what was going on (Sharon, woman with multiple myeloma).

As described by Sharon, the process of sharing information as a team can involve independent tasks. Regardless of whether information was gleaned individually or together, a perception that one’s partner participated in the flow of information at some level, be it through expressed interest, listening, processing, or gathering information, was said to increase confidence to deal with cancer-related information and decision-making together as a team.

On the contrary, a patient’s or a partner’s disinterest in cancer-related information was troublesome for dyadic efficacy.

I think one thing for us that set the tone early on was I found that I had a very strong desire to know a lot of details about cancer and numbers and survival rates and all that kind of information and I blurted out one statistic to my husband one evening while we were talking, I told him this is the ten year survival rate for da da da da da and he just looked at me and said ‘I don’t want to know that at all…’ No desire to know. I think for us as a couple, that set the stage as to what the communication was like with my husband around the cancer itself (Louise, woman with multiple myeloma).

One partner also shared about the obstacle she encountered when her expressed desire to learn more about her partner’s diagnosis was in opposition to his wishes stating, “I couldn’t go behind his back and do it because it wasn’t right. So, we just kind of muddled through” (Fiona, partner).

One member of the dyad’s expressed disinterest in cancer-related information posed a perceived insurmountable obstacle for perceptions of capability to work together as a team to manage the vast amounts of information given and obtained following a cancer diagnosis.

### C: Coping strategies used and evaluations made about the chosen strategy

Patients’ and partners’ (a) dominant coping strategy and (b) evaluation of partner’s coping strategies influenced dyadic efficacy.

#### C1: Avoidant coping

While no dominant coping strategy that facilitated dyadic efficacy was identified in the data, coping that was focused on deviation of attention, or, as referred to by some participants, “not thinking about it [cancer]” (Luc, man with myeloma), was a substantial obstacle for dyadic efficacy. Coping characterized in this way was labeled avoidant coping for the purposes of this description. One partner recounted her struggle with a partner’s avoidant coping as follows: “I find it so hard to see the positive side of it [cancer] and he does not see the negative or positive, he just does not even think about it so that’s hard because we go through it so differently” (Stephanie, partner). Efforts to not think about cancer were enacted through the use of denial, distraction, or minimization. Some participants appraised these avoidant coping efforts to be working effectively. Regardless of their effectiveness at the individual level, patients and partners of individuals who coped by trying not to think about cancer felt severely impeded in their confidence to manage cancer-related challenges conjointly.

#### C2: Acceptance versus disapproval of partner’s coping strategies

The way in which a patient or a partner evaluated the other’s coping strategies helped or hindered dyadic efficacy. Acceptance of the other’s coping behaviors was used to capture responses that ranged from suspending judgment to expressions of unconditional acceptance of the coping efforts made. Responses in between these extremes were also identified in which participants reported adapting to their partner’s coping behaviors or learning to tolerate differences in coping. Acceptance was particularly facilitative of dyadic efficacy as these participants described efforts to accept their partner’s coping behaviors even when these behaviors were different from their own coping preferences or what they believed to be best. Both those who reported a belief that their partner accepted their coping behaviors and those that expressed a conscious effort to accept their partner’s current ways of coping noted benefits for increased confidence to cope with cancer together as a unit. As one patient noted: “I do not even know if he agreed with everything but he just… I felt loved and I felt supported, even though I was crazy” (Kelly, woman with breast cancer). Acceptance of a partner’s coping efforts even extended to instances in which the coping strategies used were described to be less than ideal:

My temper has been at its worst going through this. I have a bad temper to start with, and it’s been so bad. I have yelled. I have screamed. I have thrown things. His patience with me during that has been nothing short of phenomenal (Joanne, woman with thyroid cancer).

Alternatively, patients’ or partners’ disapproval of the others’ coping strategies impeded dyadic efficacy. Disapproval was used to encompass responses ranging from expressed worries and concerns to a patient’s or a partner’s explicit judgments of the other’s coping behaviors. Expressions of disapproval were often accompanied by concern for one’s partner and a desire to help the other cope more effectively. Despite these good intentions, the tendency to negatively evaluate a partners’ coping efforts inhibited dyadic efficacy. Some participants described a process of fluctuating between disapproval and acceptance:

He would say, ‘It’s just like I’m taking medication for a cold but it’s stronger medication’. He couldn’t understand why this was worse than anything else. He says, ‘okay I’m sick but if I’m sick for something else, I’ll just take pills and I’ll be fine, same thing’. So, it got me frustrated because it’s not the same thing, but then I had to sort of let go because what’s the point, do I really want to try to convince him that it’s an awful diagnosis? And that his prognosis wasn’t good? Did I really have to rub it in and tell him remember, they told you it wasn’t a good prognosis, do you know what that means? I mean at one point I just decided it wasn’t necessary (Stephanie, partner).

Stephanie’s ability to learn to let go and move toward acceptance of her partner’s coping strategy provided an opportunity for dyadic efficacy to improve. These fluctuations between acting in ways that impeded or enhanced dyadic efficacy were common as patients and partners adapted to the cancer experience. Although this subtheme reflects patients’ and partners’ evaluations of individually focused coping efforts, it is the judgment of these coping efforts that influences dyadic efficacy. Those that felt accepted regardless of their adaptative or maladaptive coping behaviors expressed greater confidence to face cancer together as a couple.

### D: How changes to the division of everyday tasks and the couples’ sex life were managed

Cancer-related dyadic efficacy was influenced by a patient’s or a partner’s response to changes (a) in tasks and roles and (b) to the couples’ sex life.

#### D1: Flexibility versus lack of awareness in response to changes in tasks and roles

The most common adjustments being negotiated related to tasks and roles around the home (e.g., day-to-day chores), childcare responsibilities, work schedules or a partner’s transition to and from a caregiver role. The persistent influence of these changes in the everyday lives of the participants and their families heightened the importance of their successful navigation. The influence of changing tasks and roles also varied depending on how much change was required to the status quo of the couple.

Participants who perceived their partners to be flexible in response to changing roles and shifting needs for help expressed greater confidence to face these challenges together. Couples demonstrated flexibility in multiple ways. Although flexibility enhanced a patient’s response to change, the emphasis on flexibility was largely placed on partners. A conventional response was the healthy partner taking on tasks typically completed by the individual with cancer. Partners took on additional tasks around the home, altered work schedules, provided extra care for children and, for some, took on basic caregiving tasks (e.g., washing, dressing). Allowing for independence was also a form of flexibility that was particularly appreciated and facilitative of efficacy in the male-patient female-partner dyads in our sample. Flexibility was further demonstrated when patients and partners decided together which tasks required external help from family, friends or professionals. For example:

I think my biggest problem was lack of energy and tiredness. Fatigue was a big problem so I couldn’t do anything. That was where again, you know I counted on my husband and my father-in-law to really take over and do everything that I normally do around the house (Louise, woman with multiple myeloma).

The response of each partner to their new role as a patient or caregiver was a key moment of change that influenced ongoing confidence to navigate the cancer experience together.

When a patient or a partner demonstrated a lack of awareness for change in the allocation of tasks or roles, dyadic efficacy was impeded and participants reported personal frustration and relational conflict when having to direct a partner’s helping efforts or overtly ask for help. Sharon’s experience exemplified this obstacle:

Take the initiative. Because I do recognize that in every household one person will be responsible for these specific tasks and the other will be responsible for those tasks, right? However, when one of you is ill, the person who isn’t is going to have to pick up the slack. I found that very frustrating. It’s something as simple as a meal preparation. So, there was the stress of that and when you talk about it makes it sound like it’s so petty but when you’re actually going through it… it was so frustrating. To me that was big (Sharon, woman with multiple myeloma).

Participants who perceived reluctance in their partner’s willingness or awareness of the need for shifting task and role responsibilities felt less able and ultimately less confident to cope with these ongoing responsibilities conjointly.

#### D2: Patience in response to changes in the couples’ sex life

Patients and partners reported that responding to changes in their sex life was so integral to the couple relationship that successfully managing these changes generalized to their confidence to cope together with other cancer-related tasks and challenges. Cancer treatments, physical changes to the body, hormonal changes and the emotional toll of cancer were all noted to contribute to changes in the couple’s sex life. Participants in our sample did not describe a specific response to change in their sex life that presented an obstacle for dyadic efficacy. Conversely, patience in response to sexual changes enhanced couples’ dyadic efficacy. Changes to the couple’s sex life were multiple and varied including changes in function (e.g., dryness), pain, reduced or enhanced desire due to emotional or hormonal alterations or increased insecurity due to bodily changes (e.g., weight loss or gain, hair loss, mastectomy). As one couple recounted:

I didn’t want to be touched anymore or seen by people. I didn’t want to undress anymore. I felt like I was still grieving emotionally (Gina, woman with breast cancer)…. We worked it through together. We talked (Barry, partner).

Patience was beneficial when patients requested time to feel ready to re-engage in sexual intimacy which had commonly been slowed or stopped during active treatment. Responding with patience was also observed to be beneficial during sexual encounters when what was previously arousing for the patient had changed. Given the sensitive nature of changes to the sexual experience or changes to sexual body parts, patience created space for the couple to re-negotiate the ways they were typically sexual and explore other ways of enhancing intimacy. As Kelly (woman with breast cancer) stated: “sex has to be discussed between couples and it’s so delicate to discuss it.” Navigating changes to the couples’ most intimate interactions was indeed delicate but those who were willing to work with the changes were rewarded in their increased efficacy to face these and other cancer-related challenges together as unit.

### Facilitator and obstacle characteristics

Two essential elements should be noted in order to accurately understand the facilitators and obstacles to dyadic efficacy discussed here. Facilitators and obstacles were fluid with respect to time and domain. It would be inaccurate to classify participants’ global experiences as either facilitative or obstructive of dyadic efficacy. Every participant in our sample described behaviors that would facilitate and others that would restrict dyadic efficacy. Patients and partners also described fluctuations in their facilitative or restrictive behaviors over time. Despite these fluctuations over time and between domains, it was evident that some patients and partners commonly embodied more facilitative dimensions than obstacles and vice versa.

## Discussion

Placing dyadic efficacy in the broader research context, a perception of cancer as a shared stressor may form the very foundation on which cancer-related dyadic efficacy is built (Kayser et al., 2007). The theme outlined in this paper related to togetherness is akin to what others have discussed as ‘we-ness’ and may indicate that patients’ and partners’ sense of identity as a couple (shared or otherwise) influences their confidence to cope with cancer conjointly (Fergus and Reid, 2001; Kayser et al., 2007). Skerrett (2015) posited that a couples’ sense of ‘we-ness’ fosters relational resilience. Our results suggest that enhanced cancer-related dyadic efficacy may be an additional dimension of resilience connected to couples’ sense of togetherness or ‘we-ness’. Participants in our sample also discussed a stable couple relationship as foundational for dyadic efficacy. The importance of patients’ and partners’ appraisal of the couple relationship was also consistent with previous research in which higher dyadic efficacy among couples coping with rheumatoid arthritis was associated with higher relationship functioning (Sterba et al., 2007). The facilitative or restrictive effects of relationship functioning on dyadic efficacy was also consistent with Bandura’s theoretical assertion that efficacy expectations are influenced by past mastery (Bandura, 1977). Participants in our study who described higher levels of dyadic efficacy reported a history of successful conjoint coping. As an efficacy expectation, cancer-related dyadic efficacy represents patients’ and partners’ appraisal of their ability to cope conjointly. In a recent literature review, Chen and colleagues (2021) concluded that “dyadic processes, especially communication, were found to be significantly associated with dyadic outcomes for both members of a cancer couple” (p. 13). The results of the current study suggest that there may also be meaningful connections between couples’ efficacy appraisals of their joint coping efforts (dyadic efficacy) and relational outcomes (i.e., relationship satisfaction).

Several obstacles to dyadic efficacy limited the couples’ ability to share in the cancer experience together. Some of these behaviors were not only viewed as obstacles to higher levels of dyadic efficacy but as barriers that restricted the possibility for confidence in a shared response. Two obstacles, namely lack of communication and use of avoidant coping strategies, appeared detrimental to dyadic efficacy regardless of congruence with or acceptance by a partner. These obstacles share similarities or are consistent with previously identified constructs—lack of engagement, lack of emotional disclosure and avoidant coping—found to have a negative relational impact on couples coping with cancer and to make joint coping efforts less likely (Regan et al., 2015). Our results indicate that avoidant patterns exert similar restrictive effects on couples’ perceptions of their capability to cope together (dyadic efficacy) as they do on couples’ use of joint coping efforts (dyadic coping).

Attending to participants’ descriptions of their individual behaviors as well as the interactions between patient and partner behaviors enabled the identification of obstacles and facilitators that occurred at the individual and the couple-level. Communication patterns and the management of cancer-related information became obstacles to dyadic efficacy when preferences diverged. Although participants discussed the benefits of open communication, these patterns were only beneficial for dyadic efficacy when understood in the context of within-dyad preferences. For example, the prescription for more open communication would not be facilitative of dyadic efficacy when one member of a dyad preferred to limit their communication about cancer to practical concerns. It would be beneficial to further explore how the influence of communication patterns on dyadic efficacy might diverge depending on the topic being discuss (e.g., practical matters compared to intimate concerns). Similarly, dyadic efficacy was obstructed when one member actively sought cancer-related information and the other chose to limit or avoid information about cancer. Badr (2017) has previously called for more nuanced advancement in couples’ communication research that extends beyond a global recommendation for couples to enhance their open communication about cancer. Our results support this assertion and suggest that a within-dyad perspective is needed in order to accurately account for and make recommendations regarding couples’ optimal communication patterns. In related research, early investigations of dyadic coping, social support and information-seeking focused on congruence and emphasized the importance of fit between a patient’s and a partner’s behaviors (Revenson, 2003; Barnoy et al., 2006; Regan et al., 2015). Broadly, similar or complimentary styles were associated with better psychological adjustment to cancer while divergent styles were associated with poorer adjustment. The importance of congruence in our sample was limited to patients’ and partners’ communication. Apart from avoidant coping, congruence of coping styles was not described to be influential for dyadic efficacy. Dyadic efficacy was enhanced when patients and partners allowed for and accepted similarities or differences in coping. Provided the couple perceived themselves to be coping together toward a shared goal, the congruence of their coping style was not believed to influence dyadic efficacy expectations.

Participants’ descriptions of restricted communication due to an effort to protect their partner were consistent with protective buffering which has been defined as: “hiding one’s concerns, denying one’s worries, concealing discouraging information, preventing the patient from thinking about the cancer, and yielding in order to avoid disagreement” (Hagedoorn et al., 2000b, p. 275). Protective buffering has been well-examined among cancer patients and their partners with higher protective buffering behavior associated with increased psychological distress (Kuijer et al., 2000; Hagedoorn et al., 2000a; Manne et al., 2007) and poorer relationship satisfaction (Langer et al., 2009). Our results suggest that the detrimental effects of protective buffering may also extend to couples’ cancer-related dyadic efficacy.

A couples’ ability to negotiate role changes has long been a focus of clinical work in psychosocial oncology. Supporting these transitions is essential because a couples’ ability to successfully navigate role changes following a cancer diagnosis has been associated with relationship satisfaction and patients’ and partners’ experience of psychological distress (Manne and Badr, 2008; Ussher et al., 2011). Research examining relational changes in practical (e.g., roles) or intimacy domains have commonly focused on couples’ patterns of communication (e.g., Manne et al., 2010). In contrast, participants in the current study discussed the importance of flexibility and patience in response to changes in these types of domains. In addition to considering communication patterns, it may be beneficial to assess the extent to which patients’ and partners’ hold rigid perspectives on their roles and their ability to navigate change with patience.

## Limitations and future research

The focus groups conducted in the present study were smaller than what has typically been recommended in the literature (Wilkinson et al., 2004). Factors including difficulties coordinating participants’ schedules and no shows in response to poor weather and feeling ill reduced the anticipated size of the focus groups and led to what Krueger and Casey (2009) termed, mini-focus groups. Further, two focus groups included only one complete dyad and may be better referred to as an in-depth couple interview. Although the groups did not meet optimal size recommendations, the sample held sufficient “information power” (Malterud et al., 2016, p. 2) to generate meaningful themes within this new topic of study. As described by Malterud et al. (2016), higher information power lessons the demand on sample size. The information power of the current study was bolstered by the narrow study aim limited to dyadic efficacy influences and the rich quality of dialog that was facilitated within each of the mini-focus groups. Despite this, it bears repeating that our approach to this study was rooted in the social constructivist paradigm and we would be remiss to fail to acknowledge the ways in which additional themes may have been co–constructed through the additional interactions made possible within a larger focus group (Stake, 1995; Gergen, 2009). In addition to focus group size, future researchers might consider incorporating initial interviews with each individual or couple prior to focus group participation (Lambert and Loiselle, 2008). Given the conceptual complexity of the dyadic efficacy construct, participants may benefit from the opportunity to begin formulating their thoughts and opinions on the topic prior to engaging in the group dynamic.

The current study was limited by the constraints of the secondary analysis of data approach that was used. Participants were not asked directly about what influences their confidence to cope with cancer together. A more direct inquiry may have elicited additional descriptions that were not present in this data set. It would be beneficial to corroborate and refine the results presented here with a follow-up study that inquires directly about participants’ perspectives on what enhances or impedes their confidence to cope with cancer conjointly. It may also be beneficial to limit the inclusion criteria to the participation of complete dyads. Our inclusion of patients or partners alone did not allow for a systematic consideration of intraindividual differences in dyadic efficacy within couples. Although the influence of communication patterns on dyadic efficacy was situated within the interactions of a couple, a more comprehensive focus on a dyadic level of analysis would be beneficial to include in a subsequent study on couples’ cancer-related dyadic efficacy. The inclusion of complete dyads only would have enabled a more systematic consideration of similarities and difference between patients and partners in the other themes identified here (i.e., perspectives on togetherness). The decision to allow for individual participation was designed to reduce barriers to participation particularly for those with lower relationship satisfaction who may not actively pursue joint activities with their partner. The recruitment procedures used in the current study did not allow for the evaluation of this strategy. Future researchers should consider recruitment strategies that enable an evaluation of whether a more diverse sample is recruited when allowing for either couple or individual participation. With respect to diversity, the generalizability of the current study results was limited by a lack of racial, sexual and gender diversity that is common in close relationship research. Further research exploring cancer-related dyadic efficacy would benefit from actively working to recruit a more diverse sample or limiting the sample to an underrepresented group (i.e., same-sex partnerships; Williamson et al., 2022).

### Conclusion

This first analysis of obstacles and facilitators to cancer-related dyadic efficacy capitalized on the experiential expertise of individuals with cancer and their partners. These couples provided a rich account of the ways in which their relationship appraisals, communication preferences, coping dynamics, and responses to change influenced their confidence to conjointly cope with the challenges of cancer and its treatment. These thematic results are also instructive for the design and testing of an efficacy-enhancing intervention for couples coping with cancer. Recently researchers have called for the need to identify and target relational processes important for supporting dyadic coping among couples faced with cancer (Regan et al., 2015). Cancer-related dyadic efficacy has the potential to be such a target as improved confidence to cope together is likely to encourage greater use of dyadic coping strategies.

## Data availability statement

The datasets presented in this article are not readily available because consent for open data sharing was not included when participants provided informed consent. Requests for information should be directed to danielle.brosseau@kingsu.ca.

## Ethics statement

This study involved human participants and was reviewed and approved by the research ethics Committee of the Jewish General Hospital, Montréal, Canada (protocol #14-078). The patients/participants provided their written informed consent to participate in this study.

## Author contributions

DB: conceptualization, data curation, formal analysis, funding acquisition, investigation, methodology, project administration, and writing-original draft. SP: formal analysis, methodology, supervision, writing-review and editing. BA: data curation and formal analysis. AK: supervision, resources, funding acquisition, formal analysis, writing-review and editing. All authors contributed to the article and approved the submitted version.

## Funding

This research was supported by doctoral fellowships from the Psychosocial Oncology Research Training (PORT) program and the Fonds de Recherche du Québec-Santé (FRQS). The King’s University research committee provided financial support for the open access publication fee.

## Conflict of interest

The authors declare that the research was conducted in the absence of any commercial or financial relationships that could be construed as a potential conflict of interest.

## Publisher’s note

All claims expressed in this article are solely those of the authors and do not necessarily represent those of their affiliated organizations, or those of the publisher, the editors and the reviewers. Any product that may be evaluated in this article, or claim that may be made by its manufacturer, is not guaranteed or endorsed by the publisher.
